# The first mitochondrial genomes for Pyralinae (Pyralidae) and Glaphyriinae (Crambidae), with phylogenetic implications of Pyraloidea

**DOI:** 10.1371/journal.pone.0194672

**Published:** 2018-03-27

**Authors:** Wenbo Zhu, Jun Yan, Jingrui Song, Ping You

**Affiliations:** 1 College of Life Sciences, Shaanxi Normal University, Xi’an, PR China; 2 Research Center for Eco-Environmental Sciences, Northwestern Polytechnical University, Xi’an, PR China; Sichuan University, CHINA

## Abstract

In the present study, we report five complete and one nearly complete mitochondrial genomes of the Pyraloidea including the first representatives from the Pyralinae (Pyralidae) and Glaphyriinae (Crambidae). We also conduct a comparative analysis of mitogenomic features of this group. Our results show that Pyraloidea mitogenomes evolved under a common trend found in lepidopteran mitogenomes and share several typical genomic characters. The extra conserved blocks are identified in the Pyraloidea control region, and diverse missing codons formed another unique trait within Pyraloidea mitogenome. Furthermore, we reconstruct the mitogenomic phylogeny of Pyraloidea and confirm the phylogenetic position of Pyralinae and Glaphyriinae within the Pyraloidea using BI and ML method based on multiple mitochondrial datasets.

## Introduction

As a species-rich superfamily within the Lepidoptera, the Pyraloidea comprises more than 15,576 species with a world wide geographical distribution[1]. The Pyraloidea are of particular interest because it contains a large number of notorious pest of commercial crops, forests and ornamental plants, stored foodstuffs with significant economic importance. They are of further interest because of their diverse life history adaptations including larvae with phytophagous, detritivorous, coprophagous, parasitic habits, and even aquatic habitats, has prompted the idea that pyraloids could be an ideal model for biodiversity [2, 3]. An efficient taxonomy, management, and pest control of these important moths rely on a sound and comprehensive classification and phylogeny. Initially, apart from Crambidae and Pyralidae, several other families, including the Pterophoridae, Thyrididae, Hyblaeidae, Alucitidae, and Tineodidae, were historically recognized within the Pyraloidea. With a better understanding of moth and butterfly phylogenies, the current consensus view holds that most of the families formerly included in the Pyraloidea should belong to their own superfamily, and there is strong evidence from molecular studies for the sister relationship of Pyraloidea and Macroheterocera [4]. Additionally, the monophyly for Pyraloidea and two members, the Pyralidae and Crambidae, is supported by both morphological and molecular analyses [5, 6]. And the relationships among subfamilies for Pyralidae and Crambidae has also been investigated deeply based on nuclear gene data. However, due to limited samplings only a few mito-phylogenetic analyses have been involved in subfamily-level relationships [7].

The mitochondrial genome (mitogenome) is a common and practical system for comparative genomic and phylogenetic research. Recently, owing to the improvement of polymerase chain reaction (PCR) and sequencing technology, in particular the application of next-generation sequencing, mitogenome data have soared in many animal lineages, especially insects [8, 9]. To date, over 380 lepidopteran mitogenomes have been determined, while only 30 pyraloids mitogenomes representing three subfamilies of Pyralidae and eight subfamilies of Crambidae are now available (www.ncbi.nlm.nih.gov/nuccore/). A comparative study of mitogenome evolution and phylogeny of Pyraloidea highlights the requirement for broadening taxon sampling in further studies.

In the present study, we report five complete and one nearly complete pyraloids mitogenomes including the first representatives from the Pyralinae (Pyralidae) and Glaphyriinae (Crambidae). In addition, we summarize the evolutionary pattern of pyraloids mitogenomic features including base composition, codon usages, secondonary structures of transfer RNA (tRNA) and ribosomal RNA (rRNA) genes, and control region. Furthermore, the molecular phylogeny of the Pyraloidea was reconstructed using multiple mitogenomic data, which confirms for the first time the phylogenetic position of Pyralinae and Glaphyriinae.

## Materials and methods

### Specimen collection and DNA extraction

All specimens were collected in August 2014 by a light trap at Xunyangba (33.33°N, 108.33°E), Ningshan County, Shaanxi Province, China, preserved in 95% ethanol and stored at -20°C. All these specimens were identified by Ping You [10]. For each species, Voucher specimens have been deposited in the Insect Collection (Accession Number SNU-Lep-20140017-22), College of Life Sciences, Shaanxi Normal University, Xi’an, China 710062. The total DNA was extracted using a TIANamp Micro DNA Kit (Tiangen Biotech, Beijing, China), according to the manufacturer's protocol.

### PCR amplification and sequencing

Six mitogenomes were amplified with overlapping fragments using conserved primers [11]. PCRs were performed using FastPfu Fly DNA Polymerase (TransGen Biotech, Beijing, China) as previously described [12]. After purification with PCR Purification Kit (Sangon Biotech, Shanghai, China), all PCR products were sequenced directly with a primer-walking strategy.

### Genome annotation and sequence analysis

Contiguous sequences were assembled using Staden Package v1.7.0 [13]. PCGs and rRNA genes were identified based on homologous regions of published Pyraloidea mitogenomes using the Clustal X in MEGA 5 [14]. The tRNAscan-SE [15] was used to predict tRNA genes and their secondary structures. Secondary structures of the two rRNA genes were predicted according to the models for *Paracymoriza prodigalis* [12]. The base composition and codon usage were calculated using MEGA 5.

### Phylogenetic analysis

Mitogenomic phylogeny of Pyraloidea was reconstructed based on four datasets (PCG123: 13 PCGs including all codon positions; PCG123R: 2 rRNAs, 22 tRNAs and 13PCGs including all codon positions; PCG12: 13 PCGs without third codon positions; PCG12R: 2 rRNAs, 22 tRNAs and 13PCGs without third codon positions) using Bayesian inference (BI) and maximum likelihood (ML) methods. Three species from Thyrididae (*Pyrinioides aurea*, KT337662) [16], Alucitidae (*Alucita montana*, KJ508059) and Pterophoridae (*Emmelina monodactyla*, KJ508063) [17] were selected as outgroups. Each of 37 mitochondrial gene sequences was aligned with Clustal. Considering partitioning strategy of previous studies [18], a similar partitioning scheme (tRNA genes, rRNA genes, and each codon site of PCGs) was employed for phylogenetic analysis. The optimal model (GTR+I+Γ) for each partition was selected using Akaike information criterion in jModelTest [19]. The BI analyses were implemented in MrBayes 3.1.2 [20] with four MCMC chains running for five million generations. Each set was sampled every 200 generations with a burn-in of the first 25% of steps. The ML analyses were performed using RAxML 7.0.3 [21] with 1000 bootstrap replicates.

## Results and discussion

### General features of Pyraloidea mitogenomes

Full or partial mitogenomes of the six pyraloid moths (*Endotricha consocia*, *Hypsopygia regina*, *Orybina plangonalis*, *Evergestis junctalis*, *Tyspanodes striata*, *Maruca vitrata*) were generated and deposited in GenBank (Table 1). In addition, 38 complete or nearly complete mitogenomes of the Pyraloidea were integrated into a combined dataset for conducting comparative analyses. As reported in most metazoan mitogenomes, all the pyraloid mitogenomes contained 37 mitochondrial genes including 13 protein-coding genes (PCGs), 22 tRNA and 2 rRNA genes, and a putative control region (namely A+T-rich region for insects) (S1 Fig) [8, 22]. The length of five newly sequenced complete mitogenomes fell into the range of previously reported pyraloid mitogenomes (from 14,960-bp *Glyphodes pyloalis* to 15,594-bp *Ephestia kuehniella*). The gene order of pyraloid mitogenomes was highly conserved and identical with the typical gene order of Ditrysia mitogenomes. Compared with the predicted ancestral gene order for insects, however, a gene rearrangement occurred in the tRNA cluster (trnI-trnQ-trnM) among most Ditrysia mitogenomes, which is also proved as a synapomorphy for all the Ditrysia lineages [17]. Of this rearrangement event, the tandem duplication and random loss model [23] appears to be the most reasonable mechanism with the following events: firstly, the tRNA cluster (ancestral gene order trnI-trnQ-trnM) duplicated followed by the random deletion of the supernumerary genes including trnI, trnQ (the first copy) and trnM (the second copy).

**Table 1:** GenBank accession numbers of the species used in the present study.

Family	Subfamily	Species	GenBank
Pyralidae	Epipaschiinae	*Lista haraldusalis*	KF709449
	Galleriinae	*Galleria mellonella*	KT750964
		*Corcyra cephalonica*	HQ897685
	Phycitinae	*Amyelois transitella*	KT692987
		*Ephestia kuehniella*	KU877167
		*Plodia interpunctella*	KP729178
		*Meroptera pravella*	MF073207
	Pyralinae	Hypsopygia regina*	KP327714
		Endotricha consocia*	MF568544
		Orybina plangonalis*	MF568543
Crambidae	Acentropinae	*Elophila interruptalis*	KC894961
		*Paracymoriza distinctalis*	KF859965
		*Paracymoriza prodigalis*	JX144892
		*Parapoynx crisonalis*	KT443883
	Crambinae	*Chilo auricilius*	KJ174087
		*Chilo sacchariphagus*	KU188518
		*Chilo suppressalis*	JF339041
		*Diatraea saccharalis*	FJ240227
		*Pseudargyria interruptella*	KP071469
	Glaphyriinae	Evergestis junctalis*	KP347976
		*Hellula undalis*	KJ636057
	Pyraustinae	*Loxostege sticticalis*	KR080490
		*Ostrinia furnacalis*	AF467260
		*Ostrinia nubilalis*	AF442957
		*Ostrinia penitalis*	KM395814
	Schoenobiinae	*Scirpophaga incertulas*	KF751706
	Scopariinae	*Eudonia angustea*	KJ508052
	Spilomelinae	*Cnaphalocrocis medinalis*	JN246082
		*Dichocrocis punctiferalis*	JX448619
		*Glyphodes pyloalis*	KM576860
		*Glyphodes quadrimaculalis*	KF234079
		*Haritalodes derogata*	KR233479
		Maruca vitrata*	KP327715
		*Nomophila noctuella*	KM244688
		*Pycnarmon lactiferalis*	KX426346
		*Spoladea recurvalis*	KJ739310
		*Tyspanodes hypsalis*	KM453724
		Tyspanodes striata*	KP347977
Alucitidae		*Alucita montana*	KJ508059
Pterophoridae		*Emmelina monodactyla*	KJ508063
Thyrididae		*Pyrinioides aurea*	KT337662

Note

* represents the new mitochondrial genomes.

The base composition is a common genome-level character for exploring mitogenome evolution [24]. We assessed this feature of pyraloid mitogenomes by calculating A+T content, AT-skew, and GC-skew. In general, lepidopteran mitogenomes exhibit a strong bias to A and T and the negative GC-skew [8]. And our analyses confirmed that the base composition of pyraloid mitogenomes is similar to the typical trend of lepidopteran mitogenomes. Within the Pyraloidea, Crambidae and Pyralidae there was no significant difference in A+T content and GC-skew, while the AT-skew presented a distinct tendency (S2 Fig). All the Pyralidae species demonstrated the negative AT-skew (< -0.035, except for *Lista haraldusalis* -0.007), while Crambidae species showed a higher AT-skew (> -0.0249) than that of Pyralidae. Additionally, comparative analyses of A+T content and strand asymmetry at the subfamily level revealed that A+T content and AT-/GC-skew largely overlapped, suggesting that base composition evolved under an identical pattern among subfamilies from the same family. Overall, the relatively consistent patterns of base composition for the Pyraloidea mitogenomes not only reflects similar substitution pressures but appears to result from the conserved genome organization.

### Protein-coding genes and codon usage

The total size of 13 PCGs in Pyraloidea mitogenome was intermediate, ranging from 11,134 bp (*Ephestia kuehniella*) to 11,230 bp (*Chilo suppressalis*). And the average A+T content of PCGs was also similar to other moths [25], ranging from 75.1% (*Scirpophaga incertulas*) to 81.1% (*Paracymoriza distinctalis*), while the A+T content largely differed among three codon positions. The third codon position showed a far higher A+T content than that of the first and second codon positions, which is similar to other moths. Overall, all of these features of PCGs make a major contribution to the strong AT bias of whole genome.

Most of the PCGs in Pyraloidea mitogenomes possessed canonical start codons (ATN) with the exception of COI, which used CGA as a start codon. Apart from COI in most species, there were several genes with other start codons: GTG for ND3 (*Pycnarmon lactiferalis*) and TTG for ND1 (*Galleria mellonella*). Estimated among 13 PCGs, start codons of ATP6, COIII, ND4, and ND4L were the most conserved, and in contrast, COII, ND3, and ND1 held diverse start codons (Table 2). Compared with start codons, the stop codon in Pyraloidea mitogenomes was more straightforward. ND2, ATP8, ATP6, COIII, ND6, and CYTB mainly used TAA as the stop codon, and the incomplete stop codon T or TA chiefly occurred in COI, COII, ND5, and ND4. Another typical stop codon TAG scattered among COII, ND3, ND4, ND4L, and ND1.

**Table 2:** The missing codons in Pyraloidea mitochondrial genomes.

Family	Subfamily	Species	Missing codon						
Pyralidae	Epipaschiinae	*Lista haraldusalis*	AGG(S)	CGC(R)	CUG(L)				
	Galleriinae	*Galleria mellonella*	AGG(S)	AGC(S)	CGC(R)	ACG(T)	CUG(L)		
		*Corcyra cephalonica*	AGG(S)	CCG(P)	CUG(L)				
	Phycitinae	*Amyelois transitella*	AGG(S)	CGC(R)					
		*Ephestia kuehniella*	AGG(S)	CCG(P)					
		*Plodia interpunctella*	AGC(S)	GGC(G)	ACG(T)	CUG(L)			
	Pyralinae	*Hypsopygia regina*	AGG(S)	ACG(T)	UCG(S)				
		*Orybina plangonalis*	AGG(S)	ACG(T)					
Crambidae	Acentropinae	*Elophila interruptalis*	AGG(S)						
		*Paracymoriza distinctalis*	AGC(S)	CGG(R)	CGC(R)	GCG(A)	CCG(P)	UCG(S)	CUG(L)
		*Paracymoriza prodigalis*	AGC(S)	GGC(G)	CGC(R)	UCG(S)			
		*Parapoynx crisonalis*	AGG(S)	CGC(R)	CAG(Q)	GCG(A)	ACG(T)	UCG(S)	CUG(L)
	Crambinae	*Chilo auricilius*	AGC(S)	CGC(R)	CCG(P)	ACG(T)	CUG(L)		
		*Chilo sacchariphagus*	AGG(S)	CGC(R)	CUG(L)				
		*Chilo suppressalis*	GUC(V)						
		*Diatraea saccharalis*	AGG(S)	CUG(L)	CGC(R)	CCG(P)			
		*Pseudargyria interruptella*	ACG(T)	CUG(L)					
	Glaphyriinae	*Evergestis junctalis*	AGG(S)	AGC(S)	ACG(T)	CCG(P)	CUG(L)		
		*Hellula undalis*	AGG(S)	CGC(R)	CCG(P)	UCG(S)	CUG(L)		
	Pyraustinae	*Loxostege sticticalis*	AGG(S)	AGC(S)	CGG(R)	CGC(R)	UGC(C)	ACG(T)	CUC(L)
		*Ostrinia furnacalis*	ACG(T)	CUG(L)					
		*Ostrinia nubilalis*	AGG(S)	CUG(L)	CUC(L)				
		*Ostrinia penitalis*	-						
	Schoenobiinae	*Scirpophaga incertulas*	AGG(S)	ACG(T)					
	Scopariinae	*Eudonia angustea*	-						
	Spilomelinae	*Dichocrocis punctiferalis*	AGG(S)	CGC(R)					
		*Cnaphalocrocis medinalis*	AGG(S)	AGC(S)	CGC(R)	GCG(A)	ACG(T)	UCG(S)	GUC(V)
		*Glyphodes pyloalis*	AGG(S)	CGC(R)	GCG(A)	CUG(L)			
		*Glyphodes quadrimaculalis*	AGG(S)	CGG(R)	UGG(W)	CUG(L)			
		*Haritalodes derogata*	AGG(S)	AGC(S)	CUG(L)				
		*Maruca vitrata*	AGG(S)	AGC(S)	CGC(R)	CCG(P)	CUG(L)		
		*Nomophila noctuella*	AGG(S)	AGC(S)	CGG(R)	CGC(R)	GUC(V)	CUG(L)	CUC(L)
		*Pycnarmon lactiferalis*	AGG(S)	CGC(R)	UGC(C)	GUC(V)	CUG(L)	CUC(L)	
		*Spoladea recurvalis*	AGG(S)	GGC(G)	AGC(S)	CGG(R)	CGC(R)	UGG(W)	CCG(P)
		*Tyspanodes hypsalis*	AGG(S)	CUG(L)					
		*Tyspanodes striata*	AGG(S)	GGC(G)	CCG(P)				

The relative synonymous codon usage of five newly sequenced species is shown in S3 Fig. It is obvious that the usage of codons with high A/T bias is more frequent than that with G/C bias. The most frequently used codons in Pyraloidea mitogenome was identical, namely UUU, UUA, AUU (Ile), AUA (Met), UAU (Tyr) and AAU (Asn). Missing codons were constantly presented in most Pyraloidea mitogenomes with different degrees, ranged from 1 codon (*Elophila interruptalis*, *Chilo suppressalis*) to 7 codons (*Spoladea recurvalis*, *Nomophila noctuella*, *Loxostege sticticalis*, *Cnaphalocrocis medinalis*, *Parapoynx crisonalis*, *Paracymoriza distinctalis*). Generally, these missing codons showed high G/C content. Codons that were most commonly missed were those coding for the amino acid Ser, Leu, Arg. Additionally, we evaluated the lineage-specificity of missing codons, but no remarkable signal or pattern of evolution were found. On the whole, the codon usage fully reflected the A/T-preference base composition for Pyraloidea mitogenomes.

### Transfer RNA and ribosomal RNA genes

A common set of 22 tRNA genes were found in all the complete mitogenomes of Pyraloidea. These tRNA genes illustrated a consistent length and base composition among pyralids. Although most tRNAs could be folded into the typical clover-leaf structure, the exception (trnS^AGN^) existed widely in Pyraloidea mitogenomes. For trnS^AGN^, the dihydrouridine (DHU) stem was replaced by an unstable loop, which has been observed in many insect mitogenomes [26–28]. Additionally, we identified mismatched base pairs in different stems of tRNA (S4 Fig), a general feature for animal mitogenomes, and meanwhile these mismatched nucleotides might be modified during post-transcriptional processing [29].

In order to explore the evolutionary pattern of tRNA in Pyraloidea, we calculated the percentage of identical nucleotides. As shown in S5 Fig, the Crambidae and Pyralidae show similar levels of nucleotide conservation. Comprehensive analyses combining the base composition and gene arrangement revealed that the J-strand tRNAs were more conserved than N-strand tRNAs, but the identical degree did not closely link to A+T content or absolute location of mitogenome, which has been reported in other insect lineages [28]. Furthermore, S4 Fig shows that the acceptor and anticodon stem were more conserved than DHU and TψC stem, and anticodon loop also presented the highest nucleotide similarity.

Among Pyraloidea mitogenomes, the average size of rrnL (~780 bp) and rrnS (~1361 bp) is comparable to those of other moths [16,30], however a number of unique insertion sequences were identified in both of the two rRNA genes for a few species. To confirm an accurate position of these insert fragments, we predicted the secondary structures of rrnL (S6 Fig) and rrnS (S7 Fig). As observed in other insect rRNAs [11], rrnL and rrnS contain five domains (46 helices) and three domains (27 helices), respectively. According to S6 and S7 Figs, the insert fragments are mainly in domain II of rrnS and domain III of rrnL. The largest inserting-sequences were found in *Orybina plangonalis*, which included several short repeated sequences. In fact, these sequences were excluded from the stable stem region of secondary structure, so the inserted or deleted sequences of hypervariable regions did not significantly influence the function of rRNAs. In contrast, the conserved regions show a high similarity in both sequences and secondary structure. In rrnL, helices H579, H1925, and H2547 were the most conserved and stable, and helices H944, H984 and H1399 lacked variation in rrnS. It appears that rrnL evolved under a more conserved pattern than rrnS.

### Non-coding regions

In most cases, the animal mitogenome is compact and economic [22]. The largest non-coding region is generally considered to be the control region (CR). Even though the CR of Pyraloidea mitogenomes is small in size (~339.8 bp average) without any large tandem repeat sequences, it remains the longest non-coding region. The CR regulates the replication and transcription of mitogenome, and many conserved blocks (CBS) were considered to play a key role in the function of CR [31]. In insect mitogenomes, the CBS are diverse in different lineages [31, 32]. In lepidopteran mitogenomes, four conserved elements have been found in nearly all the Ditrysia species, i.e. ATAGA (CBS-1) followed by a large poly-T stretch, microsatellite structures (AT)n, and a poly-A stretch, though only three of them were identified in Pyraloidea mitogenomes (S8 Fig), which lacked the poly-A stretch. However, comparative analyses of Pyraloidea CRs indentified two other CBS: A(T)TTTA (CBS-2) and ACCRT (CBS-3). The CBS-2 was located upstream of (AT)n, while the CBS-3 occurred at the 3’ end of the CR. It should be emphasized that a clear function of these conserved elements is uncertain and thus should be included in future studies.

In addition to CRs, two other intergenic gaps (trnS-ND1 and tnrQ-ND2) existed in all Pyraloidea mitogenomes. The first gap (trnS-ND1) contained a conserved motif ATACTAW, which is involved in regulatory functions as the binding site of the transcription termination factor (DmTTF) [33]. Alignment of the second gap (trnQ-ND2) and ND2 gene showed the relatively high sequence similarity suggesting that this gap may be the debris of duplicate ND2 genes and this duplicate event should occur before the divergence of Ditrysia.

### Phylogenetic analyses

In spite of the Pyraloidea having a large number of important pests, the molecular phylogeny of the group is still ambiguous, especially for the Crambidae. Adding mitogenomes from new subfamilies and genera provide more data to investigate the phylogenetic relationships of the Pyraloidea. The inferred phylogenetic trees based on four datasets using Bayesian inference (BI) and maximum likelihood (ML) methods showed a similar topology (S9 Fig), which is consistent with other researches basically [6]. Monophyly of the families (Pyralidae and Crambidae) and subfamilies is well-supported, as is suggested by the morphological characters [34]. Comparative analyses of the trees from four datasets revealed that rRNA genes could contribute to improving the node support values, and the third codon positions of PCGs also provided phylogenetic information. Thus, the PCG123R dataset is more appropriate for reconstructing the molecular phylogeny of the Pyraloidea.

The Pyralidae is a relatively robust group, which contains five subfamilies. A previous nuclear genes study confirmed the phylogenetic relationships at the subfamily level [6], but this differs from some morphological studies [5]. In this study, we validated a stable molecular topology: (((Pyralinae + Epipaschiinae) + Phycitinae) + Galleriinae) with high support values. The main difference between morphological and molecular results is the phylogenetic position of the Pyralinae [5,6]. The Crambidae was divided into two large lineages: PS clade (Pyraustinae and Spilomelinae) and non-PS clade (the other subfamilies) [6], and supported by our results. The placement *S*. *incertulas* in this paper is the same as in [7] and [12]; both studies placed *Scirpophaga* as sister group to the Crambinae. It differs from the nuclear gene-based hypothesis [6], where *Scirpophaga* is either sister group to the Midilinae or *Rupela* + Acentropinae. Unlike the nuclear-gene based study [12]. Neither Midilinae or *Rupela* were included in this study and may account for the results.This significant difference could also be explained by long-branch attraction [35], and could be corrected by increasing the sampling number for Schoenobiinae and by combining both the nuclear and mitogenomic data. Overall, most lineages inferred by mitogenomic data confirmed the current view of Pyraloidea phylogeny.

## Supporting information

S1 Fig The gene map of the newly sequenced mitochondrial genomes of Pyraloidea. (TIF)

S2 Fig The base composition and strand asymmetry in Pyraloidea mitochondrial genomes. (A) AT% vs. AT-Skew. (B) AT% vs. AT-Skew.
(TIF)

S3 Fig The relative synonymous codon usage of five newly sequenced species. Red codons are not presented in mitochondrial PCGs.
(TIF)

S4 Fig Predicted secondary structures of tRNAs found in *Orybina plangonalis* mitochondrial genomes. (TIF)

S5 Fig Nucleotide conservation of tRNAs in Pyraloidea mitochondrial genomes. (TIF)

S6 Fig Predicted secondary structure of the rrnL of *Orybina plangonalis*. (TIF)

S7 Fig Predicted secondary structure of the rrnS of *Orybina plangonalis*. (TIF)

S8 Fig The organisation of control region in Pyraloidea mitochondrial genomes. (TIF)

S9 Fig Phylogeny of 35 Pyraloidea species based on the maximum likelihood (ML) and Bayesian inference (BI) analysis of four mitochondrial datasets (PCG123R, PCG12R, PCG123, and PCG12). The asterisk represents that all the bootstrap support values of ML and posterior probability of BI are 100 and 1 for four datasets, respectively.
(TIF)
