# Supplementary figures and images for: The first mitochondrial genomes for Pyralinae (Pyralidae) and Glaphyriinae (Crambidae), with phylogenetic implications of Pyraloidea
Source: PLoS One. 2018 Mar 27;13(3):e0194672. doi: 10.1371/journal.pone.0194672 (PMC5870975; doi:10.1371/journal.pone.0194672)

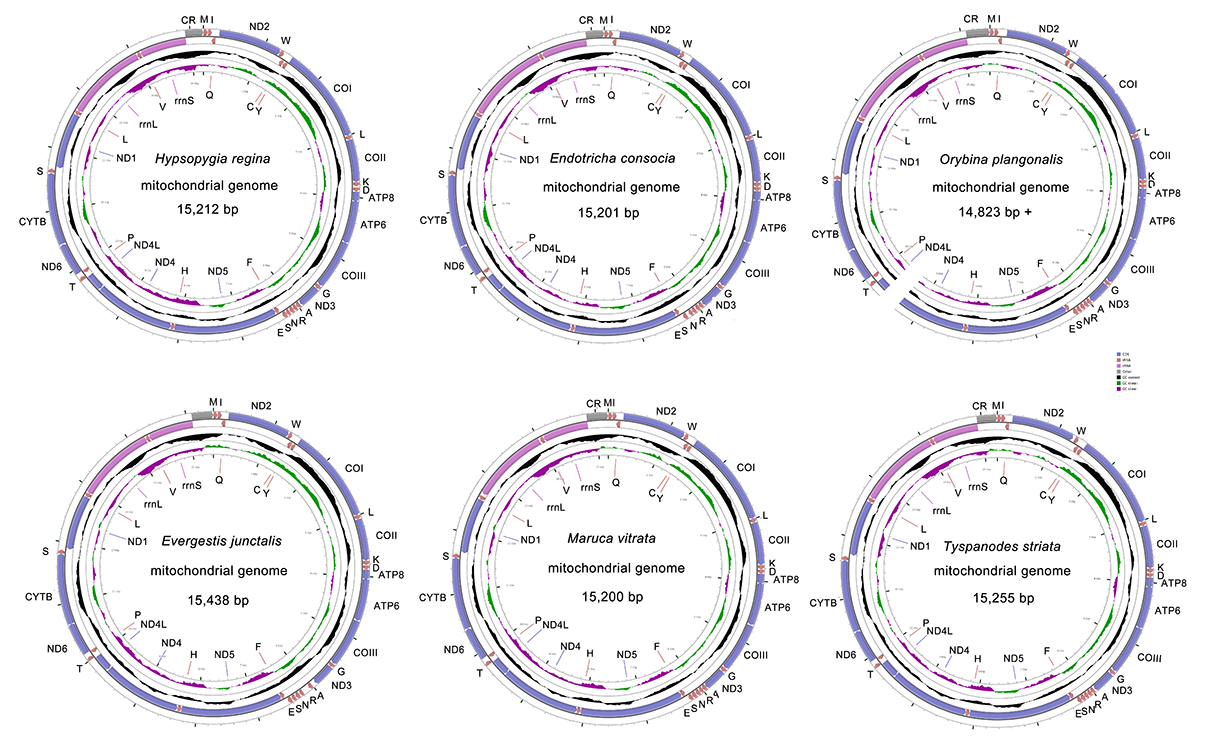

Supplement: S1 Fig — (TIF) [file pone.0194672.s001.tif]

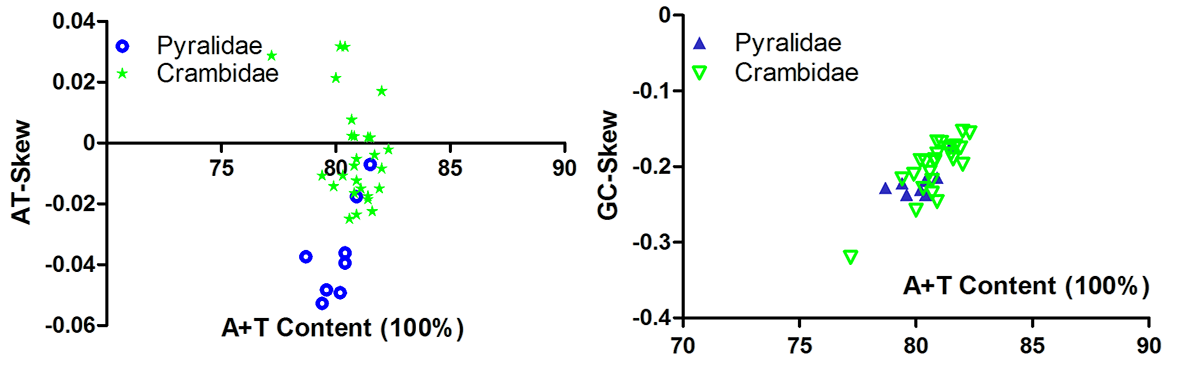

Supplement: S2 Fig — (A) AT% vs. AT-Skew. (B) AT% vs. AT-Skew. (TIF) [file pone.0194672.s002.tif]

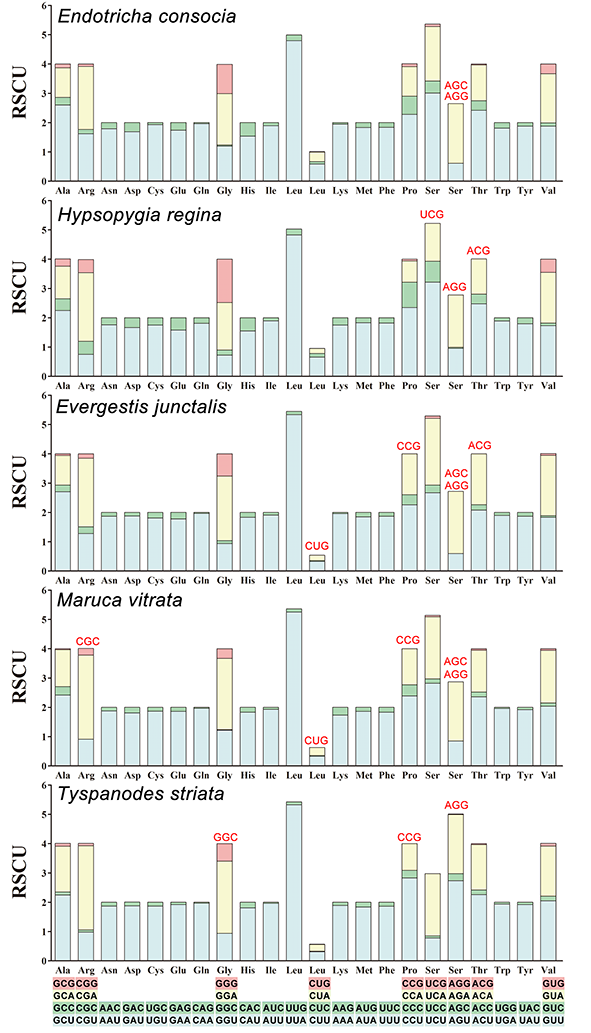

Supplement: S3 Fig — Red codons are not presented in mitochondrial PCGs. (TIF) [file pone.0194672.s003.tif]

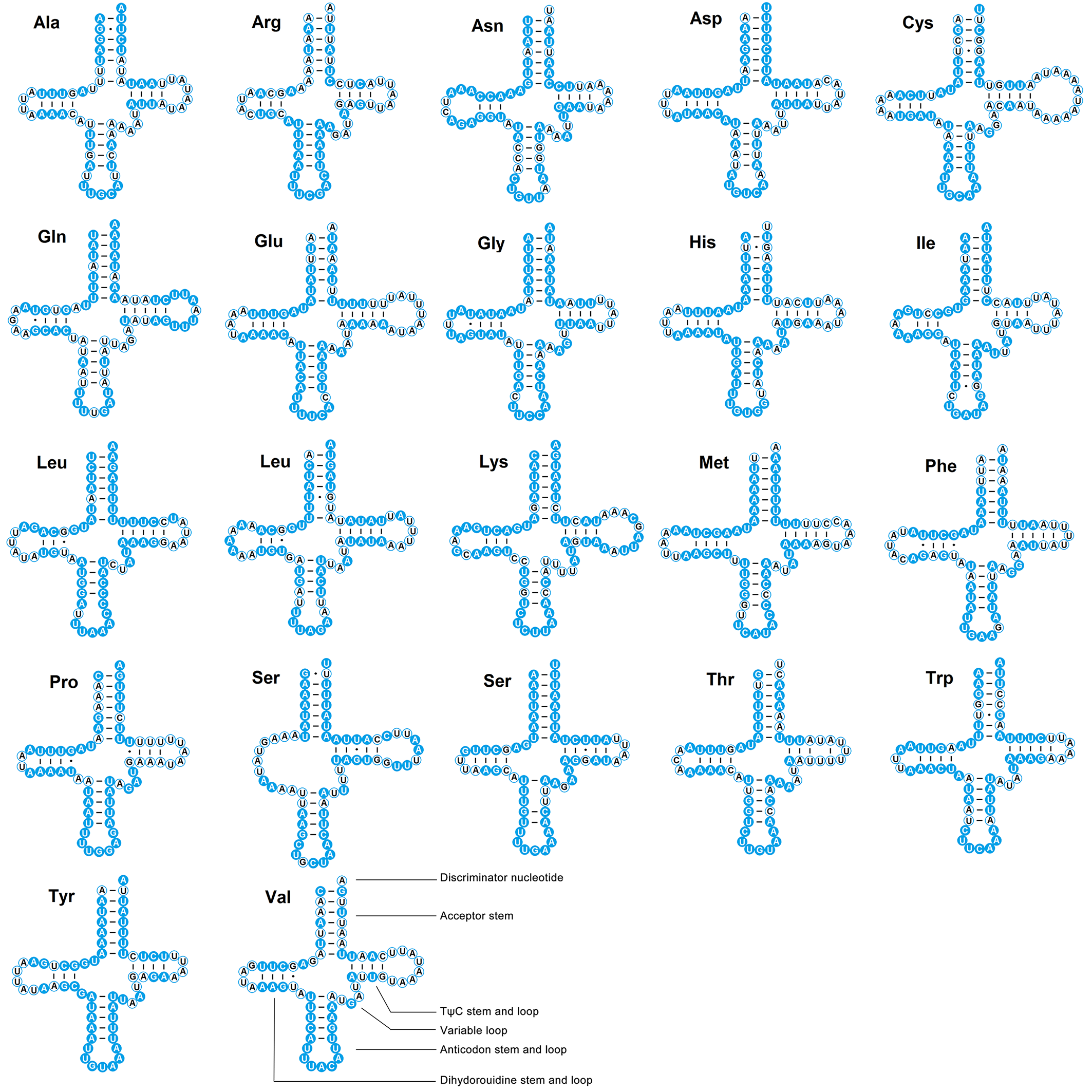

Supplement: S4 Fig — (TIF) [file pone.0194672.s004.tif]

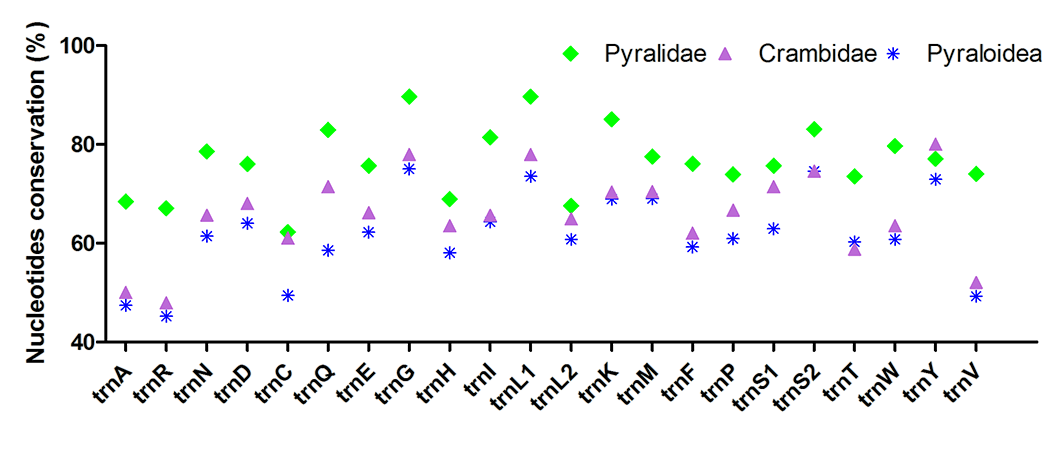

Supplement: S5 Fig — (TIF) [file pone.0194672.s005.tif]

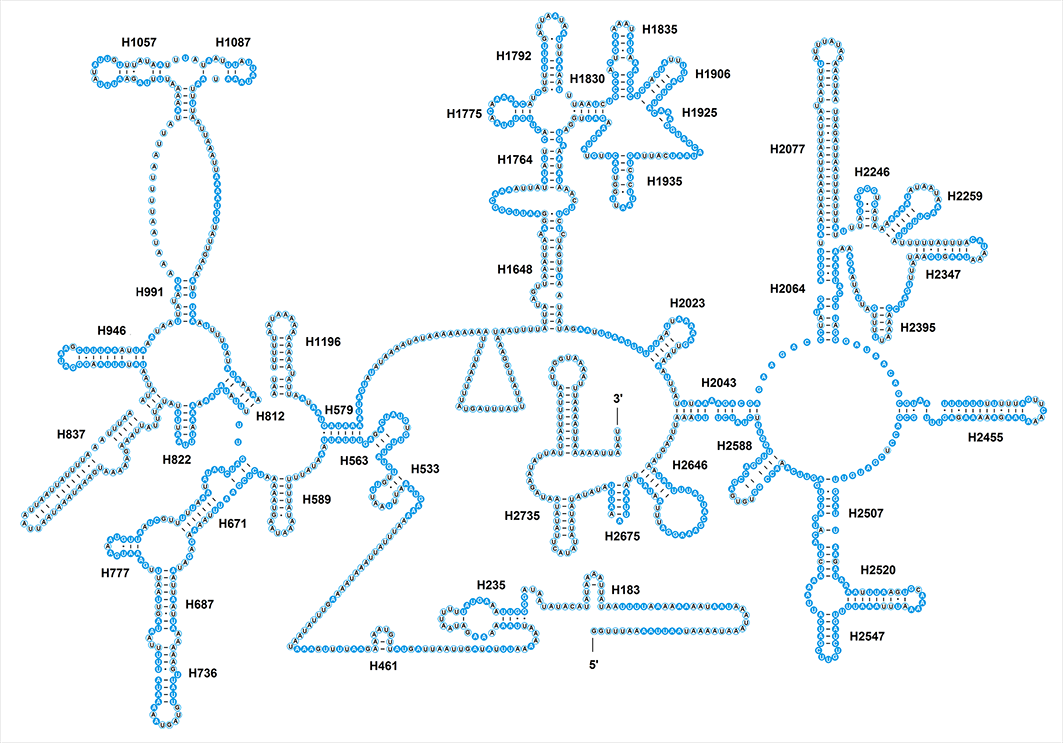

Supplement: S6 Fig — (TIF) [file pone.0194672.s006.tif]

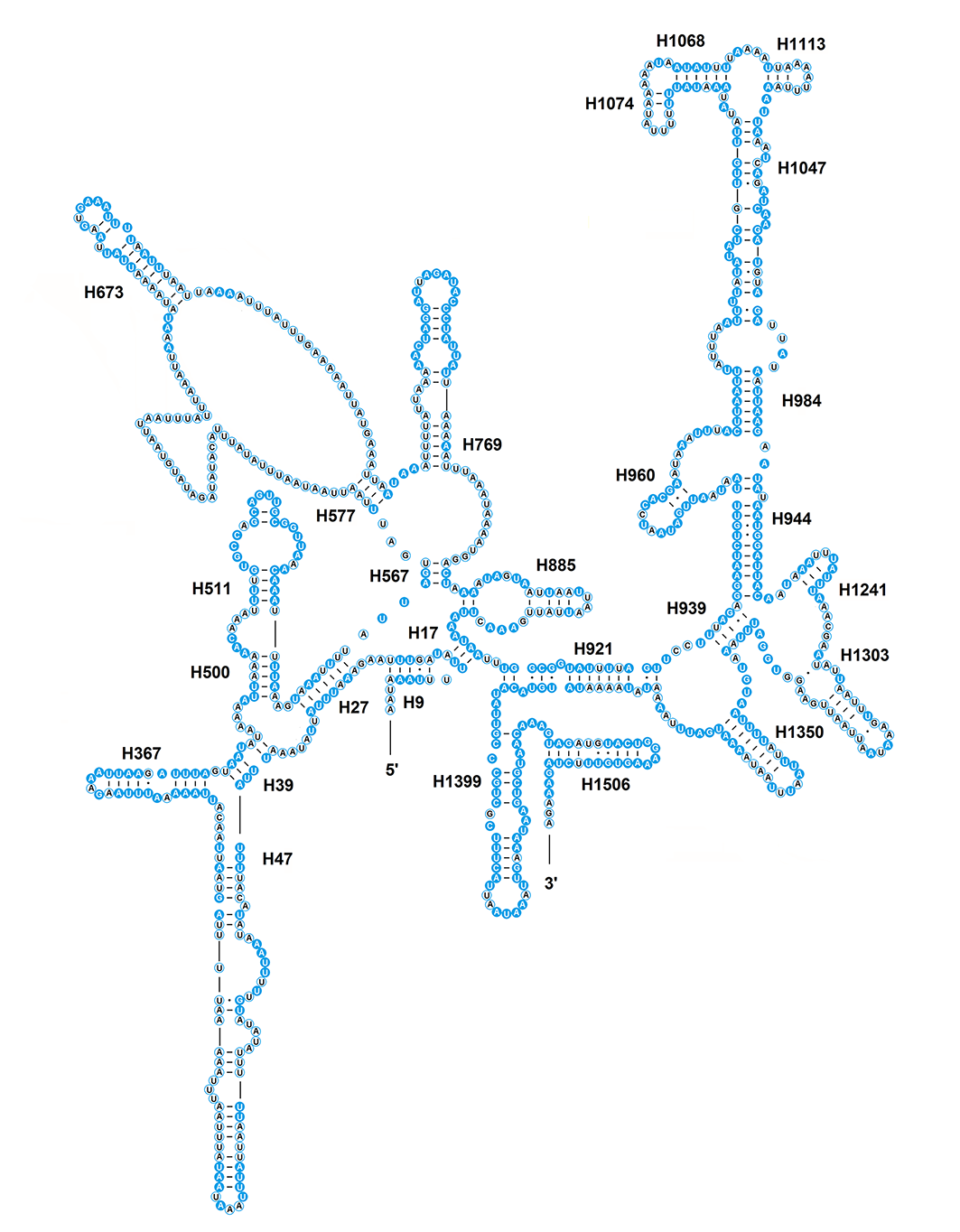

Supplement: S7 Fig — (TIF) [file pone.0194672.s007.tif]

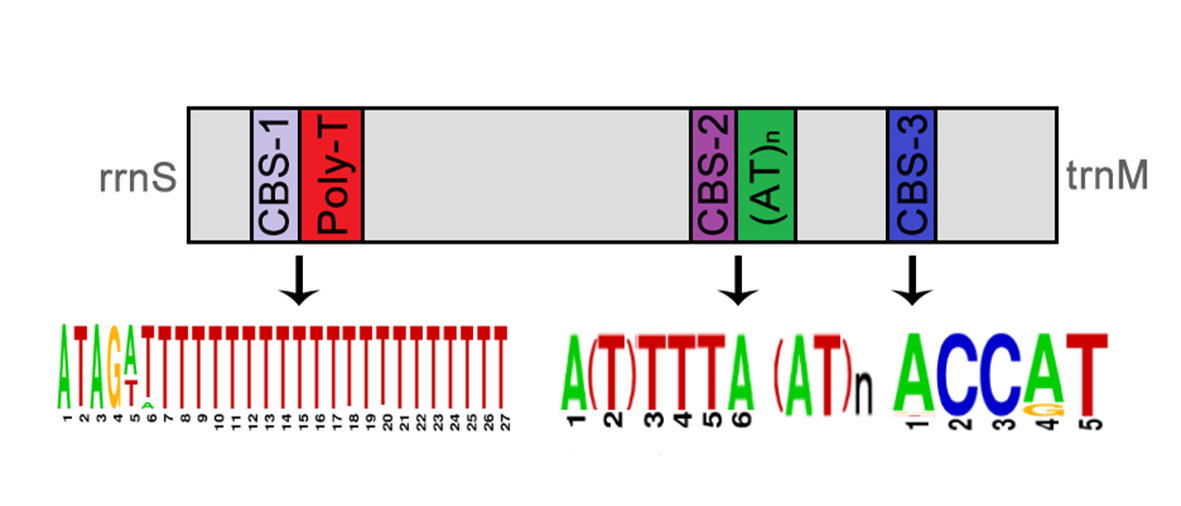

Supplement: S8 Fig — (TIF) [file pone.0194672.s008.tif]

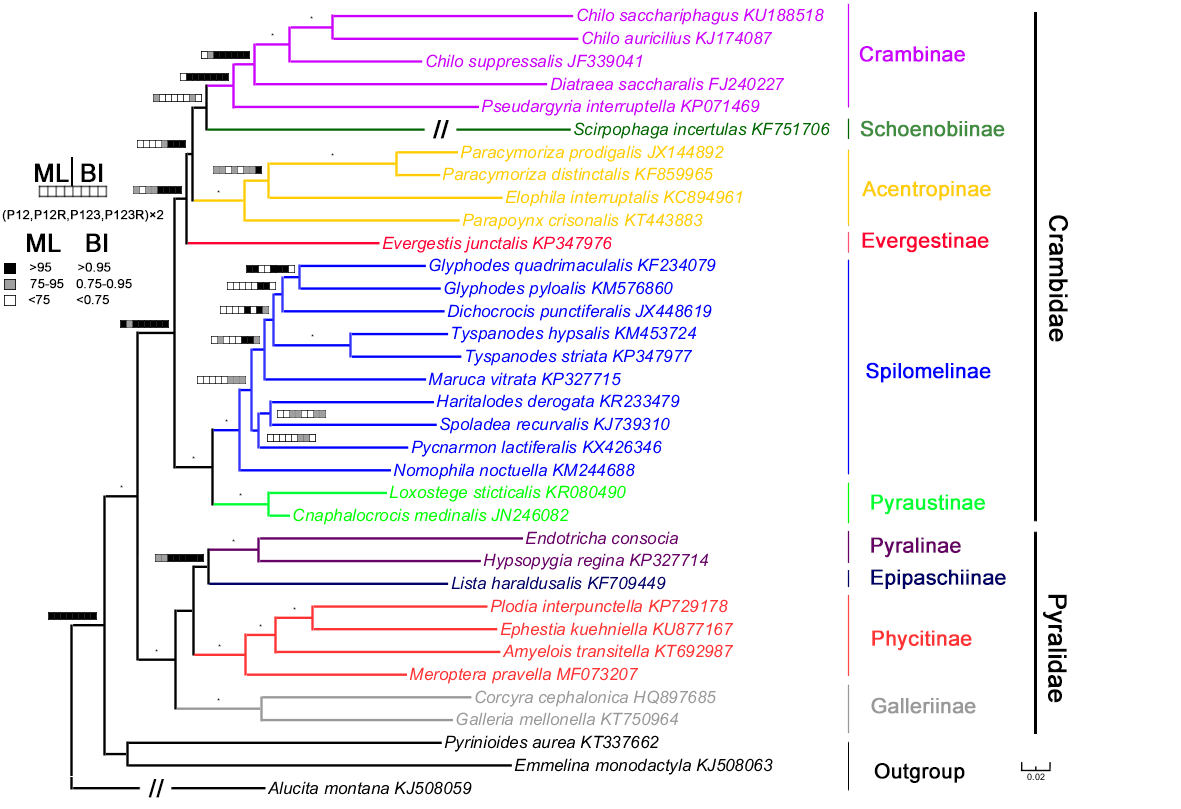

Supplement: S9 Fig — The asterisk represents that all the bootstrap support values of ML and posterior probability of BI are 100 and 1 for four datasets, respectively. (TIF) [file pone.0194672.s009.tif]
